# Galápagos and Californian sea lions are separate species: Genetic analysis of the genus *Zalophus *and its implications for conservation management

**DOI:** 10.1186/1742-9994-4-20

**Published:** 2007-09-15

**Authors:** Jochen BW Wolf, Diethard Tautz, Fritz Trillmich

**Affiliations:** 1 Department of Evolution Genetics, Institute for Genetics, University of Köln, Zülpicherstraße 47, 50674 Köln, Germany; 2 Max-Planck Institute of Evolutionary Biology, August-Thienemannstr. 2, 24306 Plön, Germany; 3 Department of Animal Behaviour; University of Bielefeld; PO Box 10 01 31; 33501 Bielefeld; Germany

## Abstract

**Background:**

Accurate formal taxonomic designations are thought to be of critical importance for the conservation of endangered taxa. The Galápagos sea lion (GSL), being appreciated as a key element of the Galápagos marine ecosystem, has lately been listed as 'vulnerable' by the IUCN. To date there is, however, hardly any scientific evidence, whether it constitutes a separate entity from its abundant Californian neighbour (CSL). In this paper, we delineate the taxonomic relationships within the genus *Zalophus *being comprised of the Galápagos sea lion, the Californian sea lion and the already extinct Japanese sea lion (JSL).

**Results:**

Using a set of different phylogenetic reconstruction approaches, we find support for monophyly of all three taxa without evidence of reticulation events. Molecular clock estimates place time to common ancestry of the Galápagos sea lion and the Californian sea lion at about 2.3 ± 0.5 mya. Genetic separation is further suggested by diagnostic SNPs in the mitochondrial and nuclear genome. Microsatellite markers confirm this trend, showing numerous private alleles at most of the 25 investigated loci. Microsatellite-based estimates of genetic differentiation between the Galápagos sea lion and the Californian sea lion indicate significant genetic differentiation. Gene diversity is 14% lower in the Galápagos sea lion than in the Californian sea lion, but there is no evidence for recent bottleneck events in the Galápagos sea lion.

**Conclusion:**

Based on molecular evidence we build a case for classifying the Galápagos sea lion (*Zalophus wollebaeki*), the Californian sea lion (*Zalophus californianus*) and the Japanese sea lion (*Zalophus japonicus*) as true species. As morphological characters do not necessarily fully reflect the rapid divergence on the molecular level, the study can be considered as a test case for deriving species status from molecular evidence. We further use the results to discuss the role of genetics in conservation policy for an organism that already is under the general protection of the habitat it lives in.

## Background

**Figure 1:**
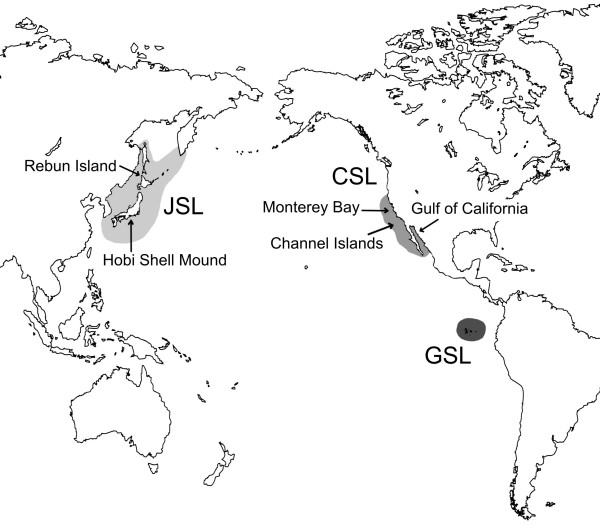
**Map of distribution for three taxa in the genus *Zalophus***. Breeding range of the Galápagos sea lion (GSL), the Californian sea lion (CSL), and putative distribution of the extinct Japanese sea lion (JSL). Modified after Heath [10] and Rice [20]. Arrows indicate sampling material used in this study.

Conservation effort is directed to biological units that can largely differ in scale ranging from a single target species to entire biocoenoses. It is nonetheless believed to be imperative in all cases that the conservation unit of interest ought to be operationally defined. On the level of single species, the concept of evolutionary significant units (ESU) has successfully been implemented to determine population units that merit separate conservation management [see [1]]. However, even in long-managed populations ecological, geographical and genetic borders, core to the ESU concept, are not always clearly delineated. This is well illustrated in the case of the endemic Galápagos sea lion, whose geographical range is well defined (Fig. 1), while it is unclear if it constitutes a unique ecological and evolutionary entity.

The Galápagos sea lion is one of the most conspicuous marine organisms of the Galápagos archipelago. Not only does it play a central role as a key predator in the Galápagos marine ecosystem. With its playful behaviour and friendly appearance it engages the sympathy of thousands of national and international visitors year by year and constitutes a keystone reference for the preservation of marine resources in the Galápagos Islands. As other marine organisms in the Galápagos it deserves special attention from a conservation viewpoint, being subject to extreme changes in food availability triggered by climatic fluctuations of the El Niño Southern Oscillation events. During such an event, mortality drastically increases and can lead to the loss of entire cohorts [2]. Population recovery may be increasingly hampered by human activities, in particular by the increasing depletion of marine resources, often in disrespect of existing regulations [[3], see also [4]]. While in the early 1960s its population was estimated at about 20.000 to 50.000 individuals and described as abundant [5,6], recent census results suggest that numbers have declined since the late 70s to approximately 14.000 individuals at present [7]. The 'IUCN Red List of Threatened Species™', which classifies species at high risk of global extinction, has categorized it as 'vulnerable' in 1996 [8]. In contrast, the Californian sister population has experienced steady growth over the last three decades [9] reaching numbers of 167.000–188.00 with a yearly pup production of about 33.000 [10]. It is classified at 'lower risk with least concern'. The JSL is considered extinct; the last credible reports date back to the late 1950s.

The taxonomic status within the three allopatric taxa of the genus *Zalophus *that so markedly differ in the degree of threat is contentious and inconsistently treated by the scientific community. Originally, the GSL has been described as a new species, *Zalophus wollebaeki *[11], but was soon relegated to sub-species level by Scheffer [12]. A representative screen of several citation databases illustrates this taxonomic uncertainty, as all of the three taxa have been treated as both subspecies and full species until today [e.g. [13,14]]. In pinniped phylogenies, the existence of the GSL is usually ignored [15,16]. Marine mammal encyclopaedias also differ in their viewpoint without specifying the reasons for their decision [10,17-20].

The aim we pursue in this study is twofold. In a first step, we address the taxonomic relationships within the three taxa of the genus *Zalophus*. We tackle the problem from the perspectives of both systematics and population biology, an approach that is more and more appreciated to confront species uncertainty in conservation [21]. The former will help to clarify the existence of characters that are shared uniquely among the specimens assigned to one taxon, and allows delineation in a purely taxonomic sense. The inclusion of the taxa into the broader phylogenetic context of all otariid species will further help to estimate genetic divergence, the degree of reproductive and historical isolation and will also contribute to a better understanding of pinniped phylogeny. The latter approach based on population genetics, describes evolutionary processes such as gene flow and helps to decide, whether it is justified to view the populations as separate evolutionary entities.

Second, we use our results to discuss the role of genetics in conservation policy for an organism that already is under the general protection of the nature reserve it lives in. We raise the question, whether better taxonomic classification is likely to affect conservation policy. We further address the important issue of genetic depauperation that can correlate with a population's resilience to introduced diseases.

## Results

### Mitochondrial DNA

#### Position of the GSL and the CLS within the otariid phylogeny

**Figure 2:**
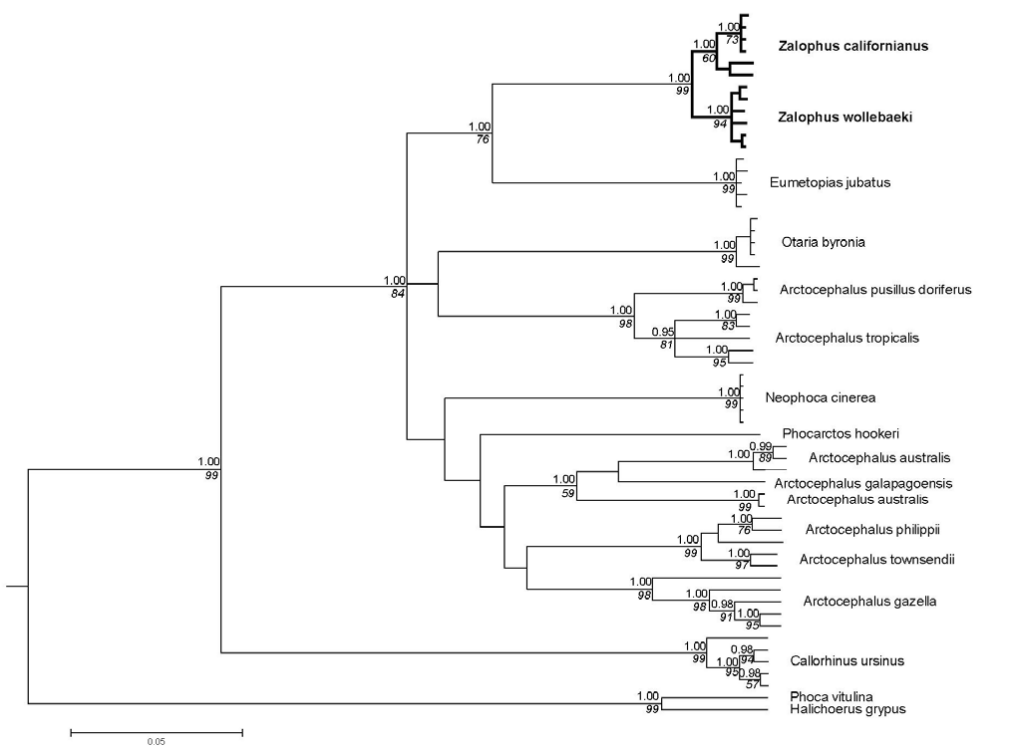
**Phylogenetic relationships of otariid seals**. 50 percent consensus cladogram of mitochondrial DNA (control region & cytochrome b) from 14 otariid and two phocid seal taxa. Posterior probability values (Bayesian clade credibilities, GTR + Γ + I model) are shown above with branches in non-italicised numbers and parsimony bootstrap support values below the nodes in italics (5000 replicates).

The phylogenetic reconstruction based on part of the mitochondrial control region and cytochrome b gene supports the major topological relationships within the otariid seals as suggested by Wynen and co-workers [15]. Sister group relationship of the two remnant *Zalophus *taxa is clearly exposed by both Bayesian and maximum parsimony analysis (Fig. 2). The Steller sea lion (*Eumetopias jubatus*) proves to be the neighbouring clade. A clear split between the CSL and GSL is supported by Bayesian analysis with maximum posterior probabilities for both clades (straight numbers Fig. 2). In the maximum parsimony bootstrap consensus, the GSL is still found to be monophyletic with high bootstrap support, while the Californian clade holds up with medium support (italic numbers Fig. 2). As the alignment with all otariid species compromises the resolution of the tree, we additionally estimated genetic divergence between the two *Zalophus *taxa separately making use of the entire available sequence information (1123 bp alignment). The number of fixed differences is considerably higher for the control region (9/623 bp) than for the cytochrome b gene (1/500 bp).

Molecular clock estimates are based on the Bayesian consensus tree, using the Phocina clade (*Phoca vitulina *and *Halichoerus grypus*) for calibration (see Methods). Minimum radiation time for the CSL and the GSL are estimated at 1.31 ± 0.43 mya and 0.58 ± 0.29 mya, respectively. Time to common ancestry of the Californian and the Galápagos sea lion is calculated to be in the range of 2.3 ± 0.5 mya.

#### Relationships within the genus Zalophus

**Figure 3:**
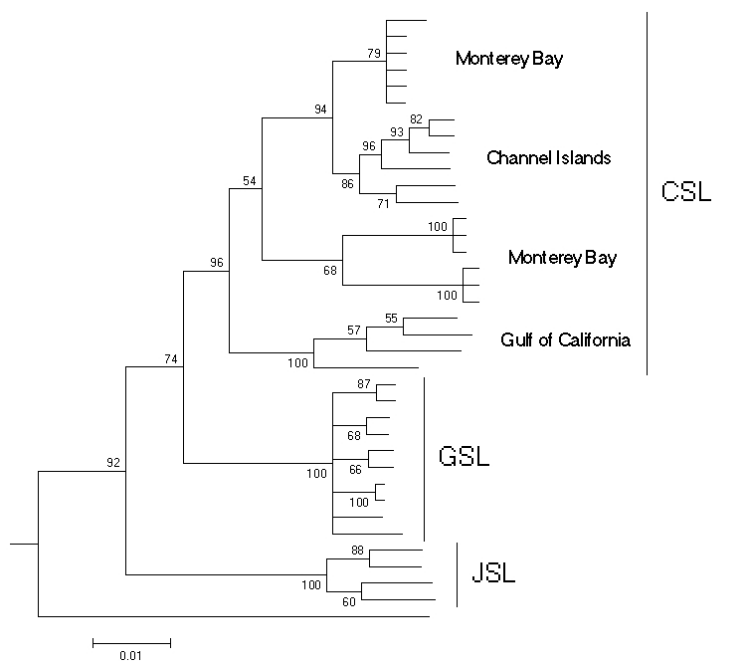
**Detailed phylogenetic relationships within the genus Zalophus**. Bayesian 50 percent consensus cladogram based on the mitochondrial control region depicting relationships within the genus *Zalophus*: Californian sea lion (CSL), Galápagos sea lion (GSL), Japanese sea lion (JSL). Posterior probability values (Bayesian clade credibilities) are shown for each node. The Steller sea lion (*Eumetopias jubatus*) has been used as the outgroup.

To account for the fact that the observed split between the GSL and the CSL might be specific for the Monterey population used in the overall otariid tree, we conducted a detailed analysis on the genus *Zalophus *including sequences of the JSL and a broader sampling of CSL populations (see Methods). Although only a relatively short sequence is available for all these samples (301 bp) the Bayesian tree identifies all three taxa as monophyletic with reasonable posterior probabilities (Fig. 3). Within the CLS divergence is high and the population in the Gulf of California stands out as a possible unit of its own.

**Figure 4:**
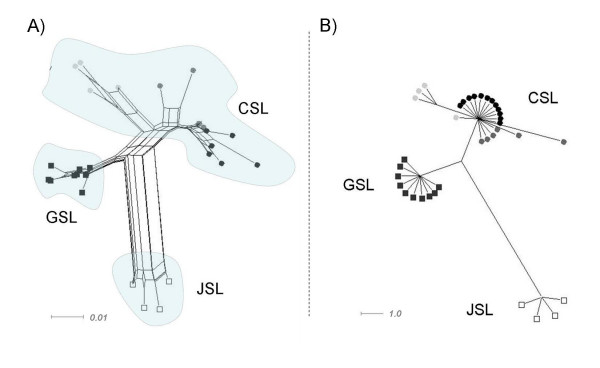
**Phylogenetic networks of the genus *Zalophus***. Phylogenetic networks based on the mitochondrial control region showing relationships within the genus *Zalophus*: Californian sea lion (CSL), Galápagos sea lion (GSL), Japanese sea lion (JSL). Geographically different populations of the CSL are colour-coded: Gulf of California (light grey), Monterey Bay (dark grey), Channel Islands (black). A) Distance based network using the Neighbor-Net algorithm. B) Parsimony based median joining network shown for a minimum split weight of two.

Phylogenetic network approaches reproduce the pattern without showing reticulation between the taxa (Fig. 4A,B). In the parsimony based median-joining algorithm (Fig. 4B) individuals from one taxon form particularly clear clusters. Again, divergence within the CSL is evident.

### Nuclear microsatellites

The mitochondrial evidence for monophyly of the CSL and the GSL is underpinned by markers of the nuclear genome. Both, the CSL and the GSL show numerous private alleles in 18 and 23 out of 25 microsatellite loci respectively (Table 1). Moreover, the two clades constitute separate population clusters in two Bayesian assignment approaches, assigning every single individual correctly with mean admixture proportions of 1.0000 in STRUCTURE and 0.9934 (± 0.0008SE) in BAPS pointing to a complete lack of exchange of individuals.

**Table 1:** Microsatellite loci. Summary of the 25 microsatellite loci used in this study and polymorphism characteristics for 1233 Galápagos sea lions (GSL) and 16 Californian sea lions (CSL).

Locus	Isolated for species	GenBank accession number	Original reference	total number of alleles	private alleles: GSL/CSL	fragment length range
ZcwA07	*Zalophus wollebaeki*	AM040044	[54]	11	2/4	280–302
ZcwB09		AM039815		7	2/2	192–204
ZcwC03		AM039819		12	8/1	256–280
ZcwC11		AM039818		14	8/0	216–248
ZcwD01		AM039817		13	3/7	234–258
ZcwD02		AM039816		15	8/2	196–238
ZcwE03		AM039821		6	1/0	224–234
ZcwH09		AM039820		5	2/1	153–165
ZcwA05		DQ836319	[56]	17	14/2	96–140
ZcwE04		DQ836324		8	3/0	125–139
ZcwA12		DQ836320		21	8/2	195–255
ZcwB07		DQ836322		6	0/1	182–192
ZcwE12		DQ836325		18	10/4	160–204
ZcwF07		DQ836326		10	4/2	138–162
ZcwE05		AM422187	first published in this article	6	1/1	198–208
ZcwG06*		AM422188		11	7/0	196–226
*Zc*CgDh4.7*	*Zalophus californianus*	AY676478	[57]	4	1/1	262–268
*Zc*CgDh5.8		AY676474		13	4/4	328–358
*Zc*CgDh7tg		AY676479		6	3/0	270–280
Hg4.2	*Halichoerus grypus*	G02090	[58]	7	3/3	150–168
Hg6.1		G02091		11	5/0	156–178
Hg6.3*		G02092		8	2/1	232–252
Hg8.10		G02093		8	3/0	172–188
SGPv9	*Phoca vitulina*	G02096		9	4/1	168–190
SGPv11		U65444	[59]	4	0/2	175–181

Loci marked with an asterisk significantly deviated from Hardy-Weinberg equilibrium and were excluded from the analysis.

Traditional population genetic measures also indicate a clear quantitative differentiation between the taxa far above random level (G-statistic: p < 0.001). All estimates of population differentiation are high, although they vary considerably. Estimators of Rst, taking allele size into account, are more than twice as high as Fst estimates based on the infinite allele assumption (SMM: Rst = 0.43; IAM: Gst' = 0.20; θ = 0.21 ± 0.03SE). This difference reflects bimodality in allele sizes and underlines the high number of private alleles in each taxon. Strongly differing sample sizes do not affect estimates of differentiation statistics. Population comparisons of CSL and ten random, equally sized sub-samples of the larger GSL database produce similar estimates (SMM: Rst = 0.40 ± 0.006; IAM: Gst' = 0.20 ± 0.002SE; θ = 0.20 ± 0.002SE). All ten comparisons indicate highly significant population differentiation, even when taking multiple testing into account. Overall, genetic distance between the CSL and GSL is an order of magnitude larger than within populations of the GSL (Rst = 0.010, Gst' = 0.009, θ = 0.009).

### Nuclear SNPs

In addition to microsatellite markers, we screened 13 unlinked loci of the nuclear genome for single nucleotide polymorphisms using genomic DNA pools of 11 individuals for each population. Out of a total of 4606 bp, we find one diagnostic difference and four polymorphic sites that are fixed in only one taxon (last two columns of Table 2).

**Table 2:** Candidate loci of single nucleotide polymorphisms between the Galápagos and the Californian sea lion. Letters used for indicating variable sites correspond to the international ambiguity code. .

Locus	GenBank accession number GSL/CSL (ENSEMBL template)	Primers 5'-3'	Expected fragment length [bp]	Aligned sequence [bp]	Base composition at variable sites	
					
					GSL	CSL
Cf4*	AM422189/AM422197 (ENSCAFG00000007422, intron 5–6)	F-ACTACGTCACGGAGGAGCTG R-GACAATGGCACGAGGTAGGT	752	328	C	Y
Cf5*	AM422190/AM422198 (ENSCAFG00000004195, intron 3–4)	F-CAAAAGGAAAAATGGCGTTC R-AGAATGCTTTTTGGCTGCTC	718	514		
Cf7 *	AM422191/AM422199 (ENSCAFG00000010325, intron 2–3)	F-GTCCTGATCGCCATGAACCT R-CACTTTATTCCCAGGGTCTCG	856	856	G	C
Cf8*	AM422192/AM422200 (ENSCAFG00000004948, intron 7–8)	F-ATCTCCCTGCAGAACACCAC R-ACCTTTTCCTGGGAACATCC	803	685		
ZcwB03^MSAT^	DQ836321^1)^	F-ATTGTACCCAAACCCAGTGC R-TCAGAATGCAATTCAGTCCAAC	383	88		
ZcwC03^MSAT^	AM039819^2)^	F-CGAAGGCCATGCTCATAACT R-GGTCAGTTATCCTGCCCAAG	303	112		
ZcwD01^MSAT^	AM039817^2)^	F-TTTACCCAGTTTGCCACCTC R-AACTTCAGAAGGGTCTAAGGAGTTC	517	152		
ZcwD03SNP	AM422193/AM422201	F-ACCCAGGAACACCTGATGTC R-GGAGGTCTCAAAACAGTGTGC	578	541	T A	Y R
ZcwD08	AM422194/AM422202	F-AACACTGCCTAGAACTTGCACA R-AGAACATTTGCCCTCAGCTC	406	406		
ZcwE03^MSAT^	AM039821^2) ^, AM422196/AM422204	F-GCACCACCTTCGGACCTAGT R-TGCCATCTTGTGTGGTGAAT	500	244	Y	C
ZcwA07^MSAT^	AM040044^2)^	F-AATGCTACCCGAACGGTTTT R-TCAATTTCCTGTCTCACCTCTAAA	464	168		
ZcwG07	AM422195/AM422203	F-GGCAAACTGTGTGATTTTAGGA R-CCTTGCCTTTCCCATAGAAAC	380	339		
ZcwH09^MSAT^	AM039820^2)^	F-GTGACAGTTAGATATTTTCCAAAGATT R-GCCTAGAAGTTTCTGATCCACCT	325	173		

^1) ^reference [56] ^2) ^reference [54]

Loci marked with an asterisk were derived from nuclear genome sequences of *Canis familiaris *and can be found in the ENSEMBL data base. Remaining loci have been specifically cloned for the Galápagos sea lion and are accessible via GenBank. Sequences containing microsatellite repeats are marked as such ^(MSAT)^. Repeat unit stretches were excluded for the analysis

### Genetic variation and bottleneck

Gene diversity is significantly lower in the GSL than in the CSL (mean over all loci: GSL 0.622 ± 0.035SE; CSL 0.720 ± 0.030SE, Wilcoxon-test: V = 49, p = 0.0118). Mean allelic richness is lower only in tendency in the GSL (GSL: 5.22 ± 0.41SE; CSL 6.04 ± 0.58SE; Wilcoxon-test: V = 69, p = 0.063). Random sub-sampling in the GSL confirms that gene diversity and allelic richness estimates are hardly influenced by differing sample sizes (bootstrapped range of mean gene diversity: 0.585–0.632, range of mean allelic richness: 5.00–5.37).

None of the loci, nor any of the sampled colonies deviates significantly from Hardy-Weinberg equilibrium. Tests for a very recent bottleneck, as assessed by statistics looking for heterozygote excess, detect no evidence for a bottleneck neither under the SMM nor under the TPM (W.-test one-sided: p = 1.00).

## Discussion

### Taxonomic status of the genus *Zalophus*

It was one goal of this study to address taxonomic relationships within the genus *Zalophus *in order to better delineate conservation units of interest. Despite of all difficulties with the species concept as such [22], to our understanding we can build a case for classifying the JSL, the GSL and the CSL as true species relative to the taxonomic practice in the entire group of the eared seals (Otariidae).

#### Japanese sea lion

Several phylogenetic reconstruction methods support monophyly for the JSL with no apparent signs of reticulation with other taxa. Our results are in line with other studies that claimed species status for the JSL based on morphological data [23,24]. As to our knowledge there is no conflicting evidence for this, we suggest to consider species status for the JSL as the null hypotheses for further studies and recommend calling it *Zalophus japonicus *hereafter.

### Californian sea lion and Galápagos sea lion

It also seems justified to reinstall species status for the GSL, as originally proposed by Sivertsen [11]. This is supported by several lines of evidence.

1) Bayesian approaches suggest reciprocal monophyly for the sister taxa (genealogical species concept). Although the amount of mtDNA divergence between the GSL and the CSL is low, it lies within the range of well established otariid species [15].

2) Molecular time estimates place the time to common ancestry at about 2.5 mya. This degree of historical isolation is not uncommonly small among pinniped species [16].

3) Ten diagnostic markers in the mitochondrial as well as one marker in the nuclear genome show that unequivocal assignment into distinct taxonomic units is possible (diagnostic species concept).

4) Evidence of clear separation between the GSL and the CSL is bolstered by population genetic analysis on the basis of microsatellite markers. Private alleles are found for nearly all loci in both taxa. While the high number of private alleles detected in the GSL could be considered as an artefact of low sample sizes in the CSL, the reverse is clearly not the case. Unique alleles found for the GSL can be regarded as real, since more than 1200 individuals from all over the Galápagos archipelago are very likely to reflect the full allelic inventory of the population. What is more, allele size distributions are often bimodal where each peak reflects one taxon. This further indicates that private alleles are more than occasional sampling dropouts. Moreover, bimodality in allele sizes also explains that Rst estimates are almost twice as high than estimates for Fst. Both estimates of genetic differentiation, as well as the assignment test point towards complete reproductive isolation (strict allopatry, biological species concept). Even if casual observations of long-distance migrants are considered, CSL and GSL ranges do not overlap [10]. This is not astounding, as populations of the CSL and the GSL are isolated by vast expanses of unproductive tropical waters.

In summary, analysis of mitochondrial and nuclear DNA suggests that the GSL and the CSL are two distinct genetic entities that deserve species status. This evidence is partly supported by behavioural studies [25,26], but contrasts with recent analyses based on morphological discrimination where a few outliers do not perfectly cluster in their group [23]. Cranial morphometric characters may thus not reflect the rapid divergence on the molecular level in the two species under study, or may be subject to convergent plasticity effects.

### Conservation management

It is widely believed that "bad taxonomy can kill", i.e. that an accurate taxonomic delineation of conservation units is of critical importance in conservation biology [27]. However, for a species that already is protected in its core range, is there anything to be gained by clarification of its taxonomic status beyond mere scientific interest? Conservation problems in the Galápagos have seriously increased with dramatically increased tourism and immigration into the archipelago over the last 10 years. Plans of long-line and drift net fishing even within the protected zone have recently been discussed seriously. Thus, the finding that the GSL constitutes a genetically unique population strengthens the position of the Galápagos National Park to argue against aggressive exploitation of the marine reserve. But even more importantly, our findings show very clearly that in case of drastic population decline, no recruitment can be expected from populations outside the Galápagos archipelago. If the GSL disappears from the Galápagos Islands it is lost worldwide.

The amount of genetic diversity is another issue of concern in conservation biology. Gene diversity in the GSL is only about 14% lower than in the CSL, but this estimate is conservative for three reasons. 1) The real diversity of the CSL is expected to be higher, as our samples were only derived from a single area (Monterey), whereas samples of the GSL cover its entire distribution. 2) Samples sizes were considerably lower in the CSL and 3) there is an ascertainment bias as the majority of microsatellites were developed for the GSL. The lower degree of genetic diversity is not due to a single recent severe El Niño event, as we found no evidence for recent bottlenecks. Instead, it is more likely a reflection of repeated bottlenecks which would lead to a generally reduced effective population size as has been suggested for the Galápagos Penguin [28]. This explanation also fits with the overall lower mitochondrial diversity of the GSL compared to the CSL (Fig. 2, 3). The lowered genetic diversity in the GSL relative to the CSL may be of concern, as reduced lowered genetic diversity can negatively affect resilience to environmental challenges and can correlate with increased disease susceptibility [29]. Taking into account that sea lion colonies partly overlap with ever growing human settlements and sea lions virtually come into people's houses and physically interact with humans and dogs, an immediate threat to a population with low genetic diversity is not a far-fetched scenario. This danger is particularly acute, as distemper virus and possibly also rabies (non-confirmed case in Isabela) that are known to ravage pinniped populations have been documented in dogs (M. Cruz, S. J. Goodman, A. A. Cunningham, personal communication). Management decisions reinforcing the separation between humans, feral or domestic carnivores and sea lions are urgently needed. Even vaccination programs on the dog population in proximity to sea lion colonies as e.g. in San Cristobal, Santa Cruz and Isabela might be advisable.

## Conclusion

Based on molecular evidence we suggest treating the Galápagos sea lion, the Californian sea lion and the Japanese sea lion by the name of species *Zalophus wollebaeki *and *Zalophus californianus *and *Zalophus japonicus*, respectively. We point out that -contrary to recent practice – all three species should be included in future studies on pinniped phylogeny. The strong divergence within the Californian sea lion further calls for a diligent analysis of the Californian clade [see also [30]].

Regarding conservation of the Galápagos sea lion we deem the general protection provided by the Galápagos National Park highly warranted. Moreover, given its small geographical range, the variable ecological conditions in its marine habitat and increasing human-induced pressure, we recommend devising more specific conservation management plans for this vulnerable species.

## Methods

### Laboratory procedures and data analyses of molecular markers

### Sample collection and DNA extraction

GSL: A total of 376 skin samples were collected from the interdigital membrane of the hind flippers of pups (< 3 months of age) at their natal rookeries and stored in 70 % ethanol. Sampling locations were uniformly spread across the Galápagos archipelago except the northernmost islands of Darwin and Wolf. Genomic DNA was extracted with the DNeasy^® ^tissue kit (Qiagen ™) and stored in Tris-EDTA buffer.

CSL: DNA samples of the CSL were supplied from locations of the Pacific Coast (Monterey Bay) central to the taxon's range containing adults (n = 5) as well as sub-adult individuals (n = 11).

The striking disproportion in sample sizes is taken into account in the subsequent analysis of microsatellite data and discussed in more detail later on. The analysis of mtDNA data is not affected since we here use only a small subset of the GSL-samples.

### Mitochondrial DNA

Part of the mitochondrial control region (625 bp) and the cytochrome b gene (500 bp) were amplified in both taxa by use of PCR with primers that were constructed on the basis of conserved regions of several mitochondrial pinniped genomes [see e.g. [16]]. After purification by ultrafiltration (Machery Nagel) PCR-products were sequenced on an ABI 3730 sequencer employing the Bigdye^® ^Terminator 3.1 cycle sequencing Kit (Applied Biosystems). A total of ten specimens of the GSL (control region: AM422165–AM422174, cytb: AM422143–AM422152) and twelve of the CSL (control region: AM422175–AM422186, cytb: AM422153–AM422164) were sequenced (see Table 3). Quality ascertainment and sequence alignment were conducted using SeqMan™ 6.1. (DNAStar Inc.). Alignments were evaluated by eye and corrected where required.

**Table 3:** Sampling locations and sample sizes. Locations of sampled rookeries, geographical coordinates and number of samples for the analysis of mitochondrial and nuclear DNA.

Taxon	Sampling location		Map coordinates	Sampled sequenced for mitochondrial DNA (GenBank Accession numbers for the control region <CR> and cytochrome b gene <cytb>)	Samples amplifying ≥ 21 micro-satellite loci
*Zalophus wollebaeki *(Galápagos Islands)	Santa Fé (Tourist beach)		0°48'18''S, 90°02'25''W	2 (*CR*: AM422165/AM422166, *cytb*: AM422143/AM422144)	39
	Española (Punta Cevallos & Gardener Bay)		1°22'07''S, 89°38'32''W		28
	Floreana (Isla Champion)		1°14'16''S, 90°23'16''W	1 (*CR*: AM422174, *cytb*: AM422152)	29
	Isabela (Villamil)		0°57'58''S, 90°57'42''W		30
	Fernandina (Cabo Hammond)		0°28'18''S, 91°36'25''W	2 (*CR*: AM422171/AM422172, *cytb*: AM422149/AM422150)	23
	Isabela (Punta Bravo) &Fernandina (Punta Espinosa)		0°09'44''S, 91°25'25''W	1 (*CR*: AM422169, *cytb*: AM422147)	27
	Pinta (Cabo Chalmers)		0°32'10''N, 90°44'20''W	1 (*CR*: AM422168, *cytb*: AM422146)	30
	Genovesa (Southwest Point)		0°18'16''N, 89°57'16''W	2 (*CR*: AM422167/AM422173, *cytb*: AM422145/AM422151)	14
	Mosquera		0°24'58''S, 90°16'42''W	1 (*CR*: AM422170, *cytb*: AM422148)	40
	Santiago (Puerto Egas)		0°14'18''S, 90°52'25''W		30
	San Cristobal (Isla Lobos, Zona Naval)		0°52'30''S, 89°36'00''W		47
	Caamaño		0°46'58''S, 90°17'42''W		30
*Zalophus californianus *(California)	Pacific Coast (Monterey Bay)	Año Nuevo Island	37°06'N, 122°19"W	10 (*CR*: AM422175/AM422176/AM422178–AM422184/AM422186; *cytb*: AM422153/AM422154/AM422156–AM422162/AM422164)	14
		Moss Landing Beach	36°47'N, 121°47W	2 (*CR*: AM422177/AM422185/85, *cytb*: AM422155/AM422163)	2
	Pacific Coast (Channel Islands)	San Miguel	See [30]	3 (*CR*: L37028/L37030/L37031)	-
		San Nicolas		1 (*CR*: L37032)	-
		Punta Banda		2 (*CR*: L37025/L37026)	-
	Gulf of California				
*Zalophus japonicus *(Japan)	Hobi Shell Mound		See [37]	3 (*CR*:AB262362–AB262364)	-
	Rebun Island			1 (*CR*:AB262365)	-

In a first step, we evaluated sister group status in a phylogenetic context including all eared seals (Otariidae). We used the sequence set from Wynen et al. [15] for both the cytochrome b gene and the control region and included a randomly chosen subset of six specimens for the GSL and six specimens for the CSL. The final alignment of the concatenated sequences of both markers consisted of 59 individuals from 16 taxa. The two phocid species *Halichoerus grypus *and *Phoca vitulina*, on which the phylogenetic tree was rooted, showed considerable longer sequences in the control region (335 bp) than the otariid seals (276 to 293 bp). No variation in sequence length was observed for the cytochrome b sequence (360 bp). Within the otariids a 24 bp region of the mitochondrial control region (bp 116–140) showed indels in basically all individuals. Sequences were arranged manually such that the number of nucleotide substitutions was minimized. Otherwise the alignment was unambiguous, several large deletions in the otariids compared to the phocid sequences could unequivocally be aligned. Final alignment length was 700 bp including gaps and 617 bp excluding gaps, respectively.

For phylogenetic reconstruction we applied maximum parsimony and Bayesian approaches. Maximum parsimony analysis was carried out in MEGA 3.1 [31] with the following settings: unweighted parsimony, close-neighbour interchange heuristic search with 500 random initial trees, including gaps as fifth character. Alignment gaps that exclusively related to the outgroup taxa were removed prior to the analysis. Confidence limits on interior branches in MP phylogeny reconstruction were estimated using bootstrap resampling with 5000 replicates [32]. Bayesian analysis was performed using MrBayes 3.1.2. [33]. To account for differential nucleotide substitution rates we partitioned the dataset (control region and codon-specific subsets of the cytochrome b gene) and chose the most complex evolutionary model of substitution rate and among-site rate variation as a starting point (GTR +Γ+I). As the more parsimonious HKY model was slightly less supported (Bayes factor comparisons), and the analysis in MrBayes is rather robust to over-paramatrization, we here report the outcome of the complex model. Results obtained from the HKY model yielded the same results. The program was run twice with four simultaneous chains and two simultaneous runs for one million generations, every 10^3 ^of which a tree was sampled. The first 50*10^3 ^MCMC steps were discarded as burn-in after which convergence of the Markov chains had long been reached. 1900 remaining trees of the two runs with highest harmonic mean likelihood were used to construct a 50% majority rule consensus.

Within the order of pinnipeds there is evidence for molecular clock-like sequence evolution [15,34], which was confirmed by Tajima's relative rate test on the two taxa in question [35] using the Steller sea lion (*Eumetopias jubatus*) as the outgroup. Molecular clock estimates were based on the work by Arnason and coworkers [16], who place the basal *Phocina *split at 4.5 mya. We used one member of each Phocina clade, *Phoca vitulina *and *Halichoerus grypus*, as our calibration point with a minimum divergence time of 4.5 mya. This estimate is better supported than the often used 'Phoca standard' [34], which assumed 2.7 mya for the Phocina split (Arnason, personal communication). Molecular distances are based on the Bayesian consensus tree and correspond to mean branch lengths of the posterior distribution. Error in divergence estimates stemming from branch length uncertainty were derived as follows: As shorter genetic distances are associated with a higher proportionate error, a calibration curve was built to adequately describe this relation and finally to predict the error associated with a given branch length. We fitted a 2^nd ^degree polynomial regression on branch length and its proportionate standard deviation in R [36] and used the predicted regression curve for calibration.

In a second step, the genus *Zalophus *including the GSL, the CLS and the JSL was examined in more detail. As populations of the CSL are known to diverge, a broader sampling across the taxon's range is desirable. To this end we used published sequences from Maldonado *et al*. [30] of the mitochondrial control region from populations further South of the Pacific Coast (Channel Islands) and from the Gulf of California. Sequences for the mitochondrial control region isolated from ancient DNA of the JSL have also just become available [37]. A detailed list of sampling locations and respective sample sizes is given in Table 3. The phylogenetic analysis had to be restricted to an alignment including 301 bp of the mitochondrial control region, as the cytochrome b information of the published CSL sequences was of bad quality, and none was available for the JSL. A phylogenetic tree rooted by the Steller sea lion was constructed with the Bayesian approach described above (using HKY as the best model of nucleotide substitution). To investigate possible reticulations between taxa, we constructed a median-joining network [38] and a distance based Neighbour-Net network [39] using the software SplitsTree4 [40]. The patterns obtained by these methods were very robust to different substitution models and were highly repeatable across other network based approaches.

### Microsatellites

Genomic DNA was genotyped for a total of 367 GSL and 16 CSL at 25 fluorescently labelled dinucleotide microsatellite loci using the Qiagen^® ^multiplex PCR Kit. To get an accurate idea on the allelic inventory of the GSL population, another 853 samples were included exclusively for the estimation of the number of private alleles (for details see Table 1). These samples stem from an ongoing behavioural study on the central islet of Caamaño [13,41,42]. Three loci (ZcwG06, Hg63, ZcCgDh4.7) deviated from Hardy-Weinberg-equilibrium (null alleles) in most of the sampled Galápagos rookeries and were excluded from the analysis. The programs STRUCTURE 2.1. [43] and BAPS 4.13. [44] were used to quantify the degree to which individuals cluster within the same taxon. Default settings were used for individual clustering in BAPS 4.13, run parameters for analysis in STRUCTURE were as follows: 10 independent runs using correlated allele frequencies and no admixture as ancestry model, burn in length 6*10^5 ^and 10^6 ^MCMC steps. Two clusters explained the data considerably better than assuming one panmictic population of both taxa (ln(P|D)_K = 1_:-23387.7 ± 0.025SE; ln(P|D)_K = 2_:-22416.1 ± 3.74SE). Structural properties within the Galápagos population are small and of no further interest in the context of this study and will be described elsewhere (Wolf *et al*., in prep.). Conventional Fst estimates [θ [45]] and the Rst estimate following Rousset [46] were used to estimate the degree of genetic differentiation between the sister taxa using Fstat 2.9.3.2. [47]. Standardized pairwise Rst distances [48] were obtained from the software Microsat 1.5d [49] and used for cluster-based tree reconstruction in the Phylip module Neighbor [50]. The G statistic proposed by Goudet [51] was used for statistical inference on population differentiation. As sample sizes markedly differed between compared populations (see above) we explicitly chose an Fst estimators that is presumably independent of sample size [45]. As sample sizes were extremely skewed, we examined the effect by random sub-sampling nonetheless. We created ten random sub-samples of 20 genotypes each from the GSL and ran the analyses again. Summary statistics are reported as means and standard errors.

Microsatellite allele frequency data were further used to uncover, whether the GSL population underwent a recent bottleneck event. We estimated gene diversity and allelic richness separately for each locus using rarefaction estimates implemented in Fstat that standardize for differing sample sizes. Differences between taxa in the two measures were assessed using Wilcoxon's matched-pairs-signed-rank tests as implemented in R [36]. We further used the software Bottleneck 1.2.02. [52], which makes use of the fact that in populations, which have experienced a recent reduction in their effective population size, number of alleles drop quicker than gene diversity. Such populations will show an unexpected high degree of gene diversity, if compared to the expected equilibrium gene diversity computed from the number of alleles [53]. The relation of observed and expected gene diversity depends on the assumed mutation model of the marker. As neutral microsatellite markers are thought to follow a stepwise mutation model (SSM) rather than an infinite allele model, we performed the analysis under the assumption of stepwise mutation and under a two phased model (TPM), which allows 10% of multi-step mutations, as recommended by the program's authors.

### Single nucleotide polymorphism

We further screened the nuclear genome for single nucleotide polymorphisms pursuing two different approaches. First, we used microsatellite clones that have specifically been developed for the GSL [for cloning procedure see [54]] including 'false positive clones' of this screen with genomic inserts devoid of microsatellite stretches. Second, we designed primers in exon regions of the dog genome (*Canis familiaris*) closing around intron sequences of appropriate size (700–850 bp). A SNP-pool detection approach was used to search for fixed differences between populations [55]. All sequencing reactions were performed on pools of DNA from each taxon including equal amounts of DNA from 11 specimens each. Sequencing was conducted as specified above. This direct sequencing of pooled DNA will somewhat underestimate rare alleles. In artificial mixing experiments we found that alleles at a frequency of 10% can still be detected, but alleles below 5% may be missed (Staubach and Tautz, unpublished). Loci, primers, expected size, length of reliable sequence and GenBank/ENSEMBL accession number are reported in Table 2.

## Authors' contributions

JBWW conceived of the study, collected most samples together with FT (see also acknowledgments), conducted the laboratory analyses, performed the phylogentic and other statistical analyses and wrote the manuscript. DT and FT contributed substantially to the content of the manuscript in their respective area of expertise and helped drafting the manuscript. All authors read and approved the final manuscript.
